# Reproducible Radiomics Features from Multi‐MRI‐Scanner Test–Retest‐Study: Influence on Performance and Generalizability of Models

**DOI:** 10.1002/jmri.29442

**Published:** 2024-05-11

**Authors:** Markus Wennmann, Lukas T. Rotkopf, Fabian Bauer, Thomas Hielscher, Jessica Kächele, Elias K. Mai, Niels Weinhold, Marc‐Steffen Raab, Hartmut Goldschmidt, Tim F. Weber, Heinz‐Peter Schlemmer, Stefan Delorme, Klaus Maier‐Hein, Peter Neher

**Affiliations:** ^1^ Division of Radiology German Cancer Research Center (DKFZ) Heidelberg Heidelberg Germany; ^2^ Diagnostic and Interventional Radiology University Hospital Heidelberg Heidelberg Germany; ^3^ Division of Biostatistics German Cancer Research Center (DKFZ) Heidelberg Heidelberg Germany; ^4^ Division of Medical Image Computing German Cancer Research Center (DKFZ) Heidelberg Heidelberg Germany; ^5^ German Cancer Consortium (DKTK) Partner Site Heidelberg Heidelberg Germany; ^6^ Heidelberg Myeloma Center, Department of Medicine University Hospital Heidelberg Heidelberg Germany; ^7^ National Center for Tumor Diseases (NCT) Heidelberg Heidelberg Germany; ^8^ Pattern Analysis and Learning Group, Department of Radiation Oncology University Hospital Heidelberg Heidelberg Germany

**Keywords:** radiomics, feature selection, machine learning, reproducibility, generalizability, multicenter

## Abstract

**Background:**

Radiomics models trained on data from one center typically show a decline of performance when applied to data from external centers, hindering their introduction into large‐scale clinical practice. Current expert recommendations suggest to use only reproducible radiomics features isolated by multiscanner test–retest experiments, which might help to overcome the problem of limited generalizability to external data.

**Purpose:**

To evaluate the influence of using only a subset of robust radiomics features, defined in a prior in vivo multi‐MRI‐scanner test–retest‐study, on the performance and generalizability of radiomics models.

**Study Type:**

Retrospective.

**Population:**

Patients with monoclonal plasma cell disorders. Training set (117 MRIs from center 1); internal test set (42 MRIs from center 1); external test set (143 MRIs from center 2–8).

**Field Strength/Sequence:**

1.5T and 3.0T; T1‐weighted turbo spin echo.

**Assessment:**

The task for the radiomics models was to predict plasma cell infiltration, determined by bone marrow biopsy, noninvasively from MRI. Radiomics machine learning models, including linear regressor, support vector regressor (SVR), and random forest regressor (RFR), were trained on data from center 1, using either all radiomics features, or using only reproducible radiomics features. Models were tested on an internal (center 1) and a multicentric external data set (center 2–8).

**Statistical Tests:**

Pearson correlation coefficient *r* and mean absolute error (MAE) between predicted and actual plasma cell infiltration. Fisher's z‐transformation, Wilcoxon signed‐rank test, Wilcoxon rank‐sum test; significance level *P* < 0.05.

**Results:**

When using only reproducible features compared with all features, the performance of the SVR on the external test set significantly improved (*r* = 0.43 vs. *r* = 0.18 and MAE = 22.6 vs. MAE = 28.2). For the RFR, the performance on the external test set deteriorated when using only reproducible instead of all radiomics features (*r* = 0.33 vs. *r* = 0.44, *P* = 0.29 and MAE = 21.9 vs. MAE = 20.5, *P* = 0.10).

**Conclusion:**

Using only reproducible radiomics features improves the external performance of some, but not all machine learning models, and did not automatically lead to an improvement of the external performance of the overall best radiomics model.

**Level of Evidence:**

3.

**Technical Efficacy:**

Stage 2.

Machine learning is increasingly used for medical image analysis. A very common approach is radiomics, in which a large amount of predefined quantitative features are extracted from the target structure, and subsequent ML models are trained to predict respective target variables as tumor tissue characteristics, genetics, or outcome.^1^, ^2^ An extensive number of Radiomics studies can be found on PubMed to date.^3^ Despite this seemly great success of radiomics in research, there is basically no translation of radiomics into clinical practice to date. It can be assumed that one major reason for this is the limited performance of most models when applied to test data from other centers, in the following referred to as external performance. It has been demonstrated that radiomics features are highly sensitive to image acquisition parameters, both for CT^4^, ^5^, ^6^, ^7^, ^8^ and MRI,^9^, ^10^, ^11^, ^12^, ^13^ explaining one major factor contributing to the decline of performance of radiomics algorithms when applied to external data. To overcome this problem, one approach proposed by several expert recommendations^14^, ^15^, ^16^, ^17^, ^18^, ^19^ would be to isolate a subset of radiomics features which are (at least relatively) robust against different image acquisition settings. By including only such reproducible radiomics features during the model building process, it could be expected that the resulting model would perform more robustly across centers with differences in image acquisition. However, performing prospective in vivo multi‐MRI‐scanner test–retest studies is challenging due to the necessity of patient recruitment, cost of MRI scan time, and scheduling of several measurements for one patient in a reasonable timeframe. Consequently, the number of prospective in vivo multi‐MRI‐scanner test–retest studies, which have investigated radiomics feature stability is very limited, including brain^20^, ^21^ and bone marrow.^9^ Other methods to define reproducible radiomics features in absence of in vivo multi‐MRI‐scanner test–retest measurements include using a simple test–retest‐experiment at the same MRI scanner, multiple delineation experiments or feature stability analysis based on image perturbation.^22^ However, the resulting features from such experiments are not necessarily reproducible across different MRI scanners for multicentric application.

The purpose of this study was to investigate how using only reproducible radiomics features, selected by a prior prospective, in vivo multi‐MRI‐scanner test–retest‐study, would influence the performance and generalizability of radiomics machine learning models on external, multicentric data sets.

## Materials and Methods

### Study Design

**FIGURE 1:**
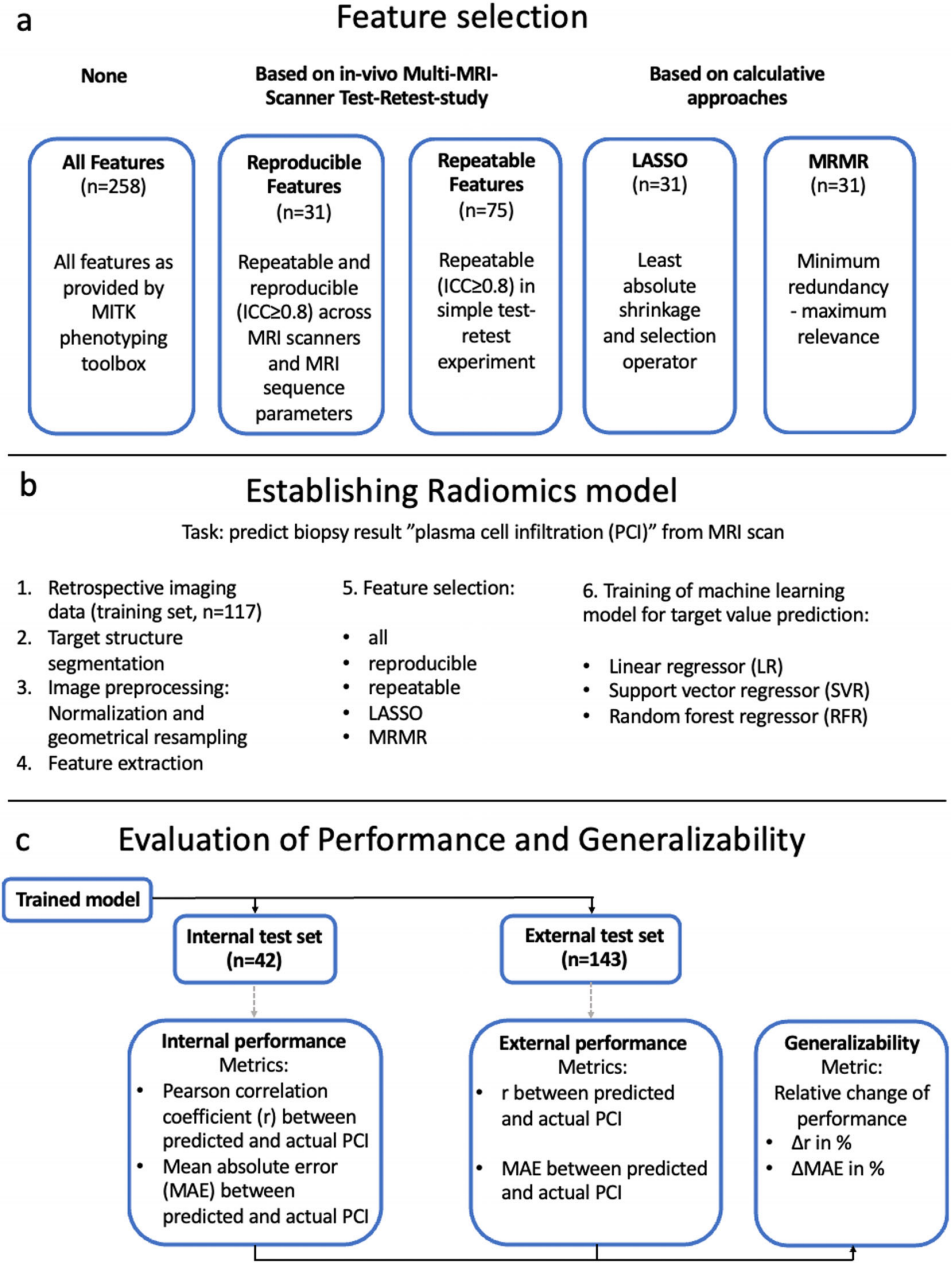
Study Design. The purpose of this study was to evaluate the influence of different feature selection methods on the performance and generalizability of radiomics models. (**a**) shows the different feature selection methods which were applied. (**b**) reports on the process of establishing the radiomics models. Especially, different subsets of radiomics features were applied, and different machine learning models were used for prediction of the target variable for each feature selection, resulting in a total of 15 different combinations of feature sets and machine learning models. (**c**) Shows an overview of the evaluation of performance and generalizability of those models. All models were applied on the internal test set and on the external test set, and correlation between predicted plasma cell infiltration by the model and actual plasma cell infiltration from biopsy and MAE between predicted and actual plasma cell infiltration, were calculated to quantify the performance of the prediction. The generalizability of each model was defined as the relative difference between the performance of the model on the internal test set and the performance on the external test set. ICC = intraclass correlation coefficient; LASSO = least absolute shrinkage and selection operator; MRMR = minimum redundancy–maximum relevance; *r* = Pearson correlation coefficient between predicted and actual plasma cell infiltration; MAE = mean absolute error between predicted and actual plasma cell infiltration; LR = linear regressor; SVR = support vector regressor; RFR = random forest regressor.

**FIGURE 2:**
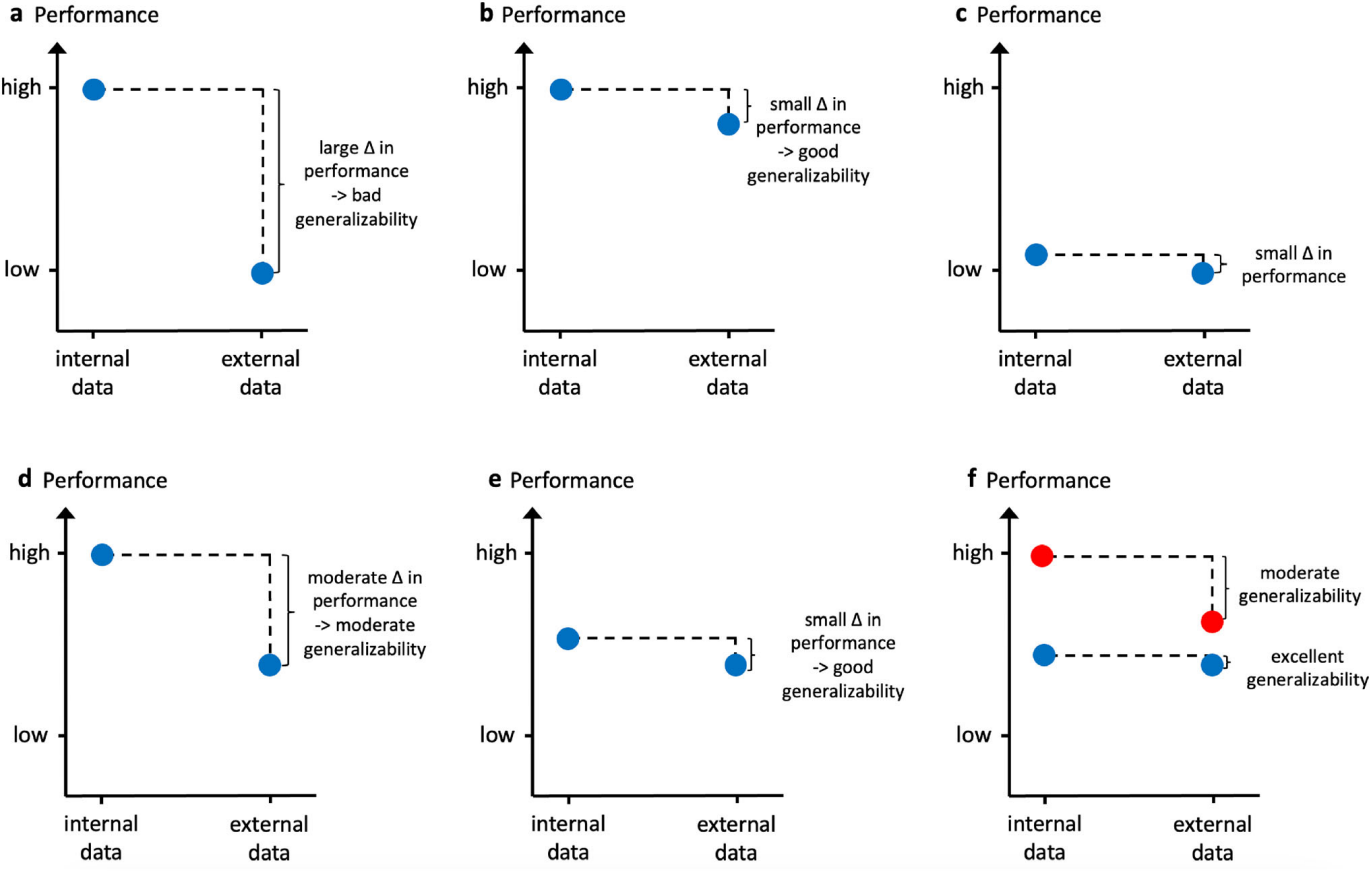
Differentiating internal performance, external performance, and generalizability: explanatory scenarios. (**a**) The model demonstrates a high performance on the internal test data, indicating a promising model. However, when applied to external data, the model does not generalize well and the external performance is low, representing a classical scenario often encountered in radiomics. (**b**) The model demonstrates a high performance on the internal test data and maintains a similarly high performance on external data, corresponding to a high generalizability, as it would be desired for large‐scale, multicentric clinical application of radiomics models. (**c**) The model demonstrates a low performance on both the internal and external test set and therefore has no clinical utility, neither at the center where it was established nor elsewhere. (**d**, **e**) When tested on the external test data, both models in d and e show the same moderate performance. In absence of an internal test set, the models would therefore seem similar, despite their striking differences regarding internal performance and generalizability. A study design with both an internal and an external test set enables to differentiate whether a limited external performance results from a low generalizability (d), or whether it stems from a generally low/moderate performance on the internal test set (e), with or without an additional problem regarding generalizability. (**f**) Even though a model shows an excellent generalizability (blue model), its external performance might still be outperformed on external test data by second model with only moderate generalizability (red model), if the second model had shown a markedly higher internal performance.

This was a retrospective exploratory study approved by the local IRB with waiver of informed consent (S‐537/2020). Different feature selection approaches, including an approach based on prior knowledge of radiomics feature reproducibility, as well as approaches based on calculative feature reduction strategies, were applied prior to training of the prediction model. The machine learning models for prediction of the target variable were trained on training data from center 1 (*n* = 117) for each of the different radiomics feature sets. The performance of the resulting models was evaluated first on an independent test set from center 1 (*n* = 42) and second on a heterogeneous external test set from seven different centers (*n* = 143). The absolute performance of the models on the internal and external test set and the generalizability of the models, defined as the relative difference between the performance on the internal and external test sets, were evaluated. A detailed overview of the study design is provided in Fig. 1. Explanatory example cases for models with different types of internal performance, external performance, and generalizability are displayed in Fig. 2.

### Prediction Task and Data Sets

The task for the radiomics models was to predict bone marrow plasma cell infiltration from pelvic bone marrow segmentations on T1‐weighted turbo spin echo images from whole‐body MRI in patients with untreated newly diagnosed multiple myeloma or the precursor stages monoclonal gammopathy of undetermined significance and smoldering multiple myeloma, as reported elsewhere.^23^ Imaging data, segmentations, and biopsy results were reused from another study,^23^ with the exception that former dataset IV from center 1 was excluded, as it contained older imaging data with lower image quality.^23^ Data were acquired between 2015 and 2021. Details on inclusion and exclusion criteria have been published elsewhere.^23^ To summarize the main inclusion criteria, all patients had a monoclonal plasma cell disorder, had undergone whole‐body MRI including a coronal T1‐tse sequence covering the pelvis, and had received bone marrow biopsy, both prior to start of systemic treatment for multiple myeloma. Images with major artifacts or large implants in the pelvis were excluded. 302 MRIs with corresponding bone marrow biopsy results from a total of 300 patients (mean age, 60 years ±10; 183 men) from eight centers were included. Table 1 reports descriptive information on the training set and the internal and external test set. The flow‐charts reporting inclusion and exclusion for this dataset have been reported elsewhere.^23^ The external test set comprises data from eight different MRI scanners (seven different MRI models) from three vendors, however only three were acquired with 3T. Additional information on the data sets and on subject overlaps with other publications^9^, ^23^, ^24^, ^25^, ^26^, ^27^, ^28^ are provided in the Data S1 in the Supplemental Material. The data reused in this study in part originated from the prospective registered trials GMMG‐HD7 (EudraCT: 2017–004768‐37) and Transregio‐79 (ClinicalTrials.gov: NCT01374412).

**TABLE 1:** Description of Study Cohorts

Data Set	Training Set (Center 1)	Internal Test Set (Center 1)	External Test Set (Center 2–8)
*n* wb‐MRIs (*n* patients)	117 (115)	42 (42)	143 (143)
Patient characteristic			
Male sex (*n*, %)	68 (59%)	24 (57%)	91 (64%)
Age in years^a^	60 ± 9	60 ± 10	60 ± 11
Female subgroup	57 ± 10	63 ± 11	61 ± 10
Male subgroup	61 ± 8	57 ± 9	59 ± 11
Disease stage			
MGUS and SMM	47	18	21
NDMM	68	24	122
Tumor load surrogates			
Plasma cell infiltration in %^b^	20 (14–40)	20 (16–32)	35 (15–60)
M‐Protein in g/L^b^	23 (13–37; 14)	23 (13–38; 6)	31 (14–41)

Descriptive information is reported for each data set.

% = percentage of this cohort; wb‐MRI = whole‐body magnetic resonance imaging; MGUS = monoclonal gammopathy of unknown significance; SMM = smoldering multiple myeloma; NDMM = newly diagnosed multiple myeloma.

^a^ Mean ± standard deviation.

^b^ Median (interquartile range).

### Imaging, Segmentation, and Radiomics Feature Calculation

Pre‐existing annotated data from a prior study was reused, and details on MRI image acquisition and segmentation are provided elsewhere.^23^ In summary, pelvic bone marrow of both hip bones was segmented on T1‐weighted turbo spin echo whole‐body MRI images. Images were normalized to the signal intensity of the piriformis muscle, as an earlier study had demonstrated that using this normalization improves the reproducibility of the majority of radiomics features in this setting.^9^ Images were geometrically resampled to a common spatial resolution (in‐plane resolution 1.3 x 1.3 mm^2^, slice thickness 5 mm, 10% distance factor).^23^ 258 radiomics features were extracted using the IBSI‐conform^29^ and validated MITK phenotyping^30^ toolbox (version 2022.4, German Cancer Research Center, Heidelberg, Germany).

### Clinical Data

All included patients had undergone routine bone marrow biopsy at the posterior iliac crest without image guidance. The percentage of plasma cell infiltration in the bone marrow was then obtained from the specimen either histologically, cytologically or both. In line with recommendations from the International Myeloma Working Group, in case of disparity between both values the higher value was used.^31^

### Feature Selection Methods

Different feature selection methods were applied prior to training of the ML models. The simplest approach is to use all calculated features. An alternative approach proposed by several expert recommendations^14^, ^15^, ^16^, ^17^, ^18^, ^19^ is to isolate a subset of radiomics features which are (at least relatively) robust against different image acquisition settings. By including only such reproducible radiomics features during the model building process, in theory it would be expected that the resulting model would perform (more) robust across centers with differences in image acquisition.

The radiomics quality score^14^ suggests to perform phantom studies on different scanners (criterion 3), and to perform test–retest scans at the same scanner (criterion 4), and then perform feature reduction based on the observed feature robustness in both aforementioned settings (criterion 5). Based on this recommendation, our group had performed a prospective in‐vivo multi‐MRI‐scanner test–retest study, in which patients with monoclonal plasma cell disorders had undergone a simple test retest at the same MRI scanner with the same MRI protocol after patient repositing (simple test–retest), as well as additional re‐scans with a second MRI protocol at the same 1.5T MRI scanner, a rescan at a second MRI scanner with the same field strength from the same vendor, and a rescan at another MRI scanner with 3.0T from the same vendor. Based on these measurements, a subset of 31 reproducible radiomics features had been identified, which had shown an intraclass correlation coefficient (ICC) ≥0.8 in all reproducibility experiments.^9^ Additionally, to mimic a scenario in which a simple test–retest experiment is performed but data on Multi‐MRI‐scanner reproducibility is absent, we performed an experiment using only the 75 radiomics features which had shown an ≥0.8 in the simple test–retest experiment in the earlier study.^9^

For comparison, we included two other, merely calculative feature selection methods. We chose least absolute shrinkage and selection operator (LASSO) and minimum redundancy–maximum relevance (MRMR) algorithms, as based on our observations these are frequently used in radiomics studies in the literature and have been demonstrated to be good choices by a large earlier study which had systematically compared 29 different calculative feature selection algorithms across 10 datasets.^32^ To eliminate a potential source of bias caused by different numbers of features between the different subsets, we applied LASSO and MRMR to also select 31 features. Further details on feature selection and a list of all feature subsets are included in the Supplemental Material.

### Radiomics Machine Learning Models for Prediction

We considered the possibility that the performance of the radiomics model might depend on the ML model used for the prediction task. We further considered that also the generalizability, and the influence that the respective features selection has on the generalizability, might depend on the ML model used for the prediction task. Therefore, we decided to include three different ML models instead of only one ML model in this study. These comprised linear regressor (LR), support vector regressor (SVR), and random forest regressor (RFR). Further details on training of the machine learning algorithms are reported in the Supplemental Material.

### Statistics

Pearson correlation coefficient *r* between predicted and actual plasma cell infiltration and mean absolute error (MAE) between predicted and actual plasma cell infiltration, were used as metrics to quantify the internal and external performance of the prediction algorithms. The relative change of both *r* (Δ*r*) and MAE (ΔMAE) between the performance on the internal and external test set were calculated as indicators for generalizability of the prediction algorithm from internal to external application. To test for differences in *r* between different models on the same data set or using the same model on different data sets, Fisher's z‐Transformation was used.^33^ To test for differences in MAE between different ML models on the same data set or the same ML models using different feature subsets on the same data set, the Wilcoxon signed‐rank test was used. To test for differences in MAE using the same model on different data sets, the Wilcoxon rank‐sum test was used. *P*‐values below 0.05 were considered statistically significant. When the term significant is used in this manuscript, this refers to statistical significance. Python (version 3.11.6; https://www.python.org/) with scipy (version 1.12.0; https://scipy.org) were used for the statistical analysis.

## Results

### Comparison of Feature Subsets Selected by the Different Feature Selection Approaches

When comparing the different feature sets selected by the respective methods (Tables S2–S5 in the Supplemental Material), it can be observed that a markedly higher proportion of texture features was selected by the calculative feature selection approaches LASSO (19 of 31 features, 61%) and MRMR (22 of 31 features, 71%) than by the reproducibility experiment (6 of 31 features, 19%) or by the repeatability experiment (21 of 75 features, 28%).

### Influence of Feature Selection on the Performance of Radiomics Models on the Internal Test Set

**FIGURE 3:**
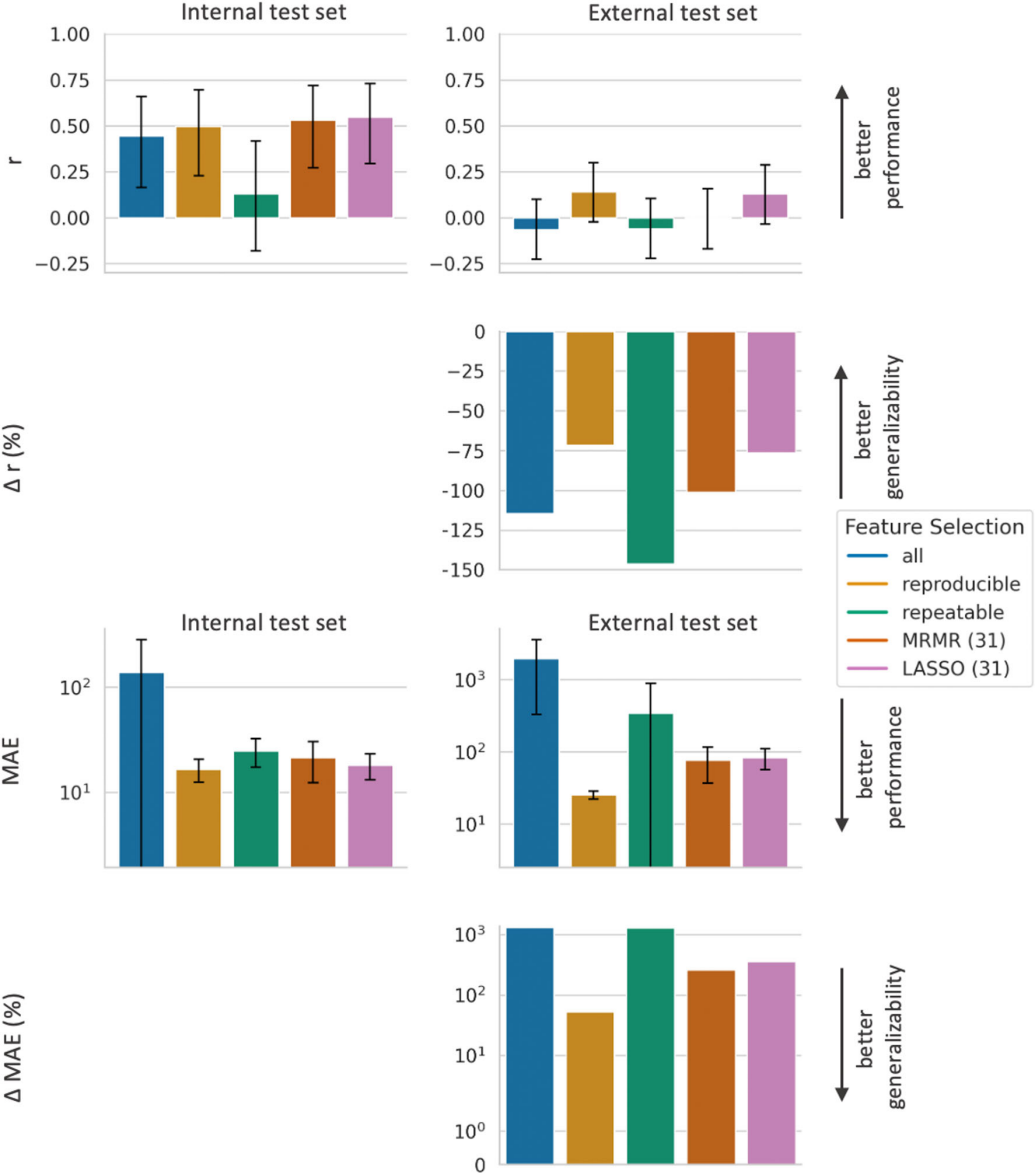
Performance and generalizability of LR models based on different feature subsets. LR models based on five different feature subsets, resulting from the five different feature selection approaches (all features, reproducible features, repeatable features, features selected by LASSO, and features selected by MRMR), are compared regarding performance and generalizability. The top row demonstrates the Pearson correlation coefficient *r* between predicted and actual plasma cell infiltration for each model‐feature‐selection‐combination, where a higher *r* indicates a better performance. The second row shows the relative decline in *r* (Δ*r* in %) between the respective model‐feature‐combination on the internal and external test set, where a less negative *r* represents a better generalizability. The third row demonstrates the MAE between predicted and actual plasma cell infiltration for each model‐feature‐selection‐combination, where a lower MAE indicates a better performance. The bottom row shows the relative increase in MAE (ΔMAE in %) between the respective model‐feature‐combination on the internal and external test set, where a lower ΔMAE represents a better generalizability. Bars indicate 95% confidence intervals. LR = linear regressor; *r* = Pearson correlation coefficient between predicted and actual plasma cell infiltration; MAE = mean absolute error between predicted and actual plasma cell infiltration; LASSO = least absolute shrinkage and selection operator; MRMR = minimum redundancy–maximum relevance.

**FIGURE 4:**
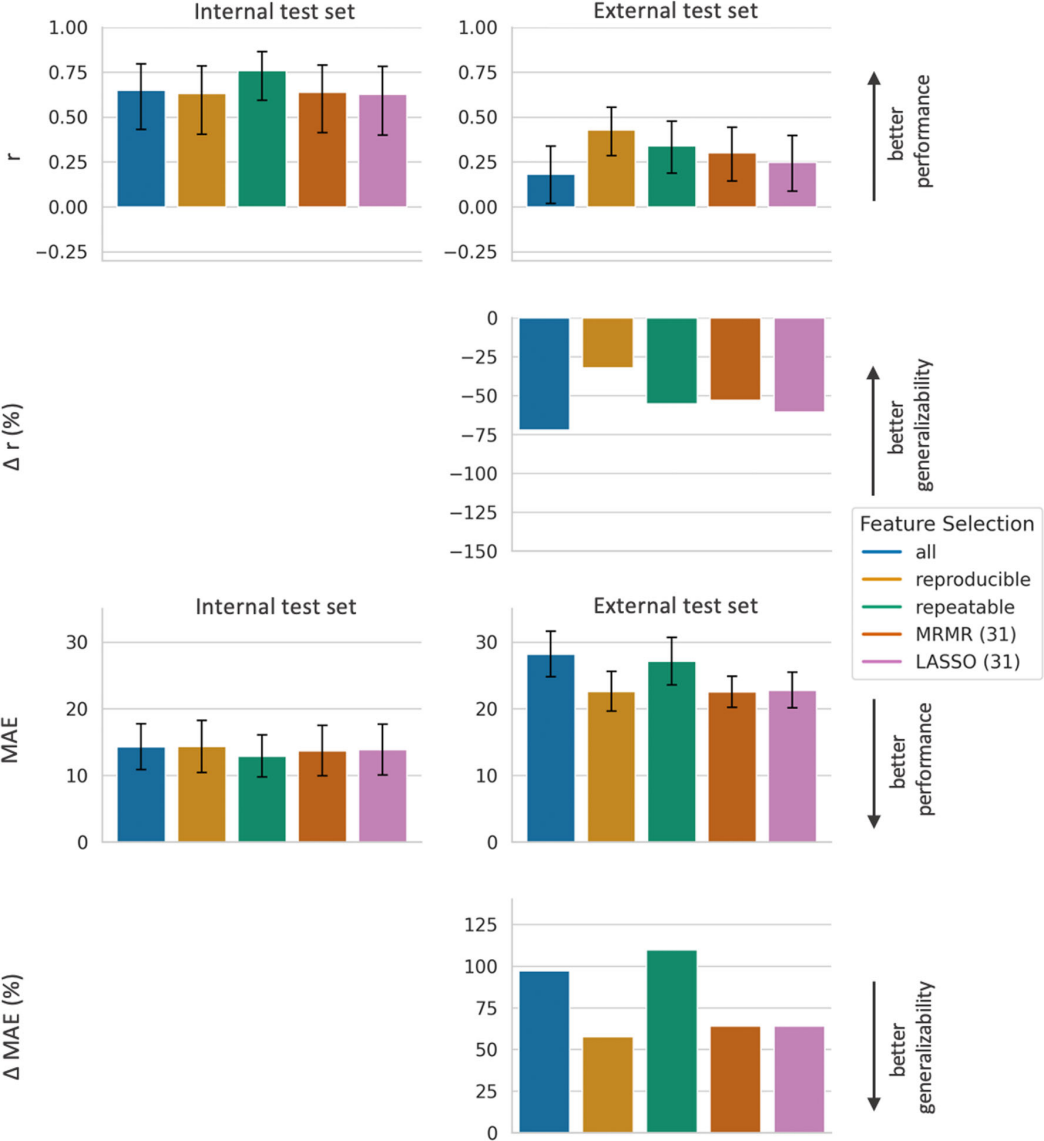
Performance and generalizability of SVR models based on different feature subsets. SVR models based on five different feature subsets, resulting from the five different feature selection approaches (all features, reproducible features, repeatable features, features selected by LASSO, and features selected by MRMR) are compared regarding performance and generalizability. For explanation and abbreviations please see legend of Figure 3. SVR = support vector regressor.

**FIGURE 5:**
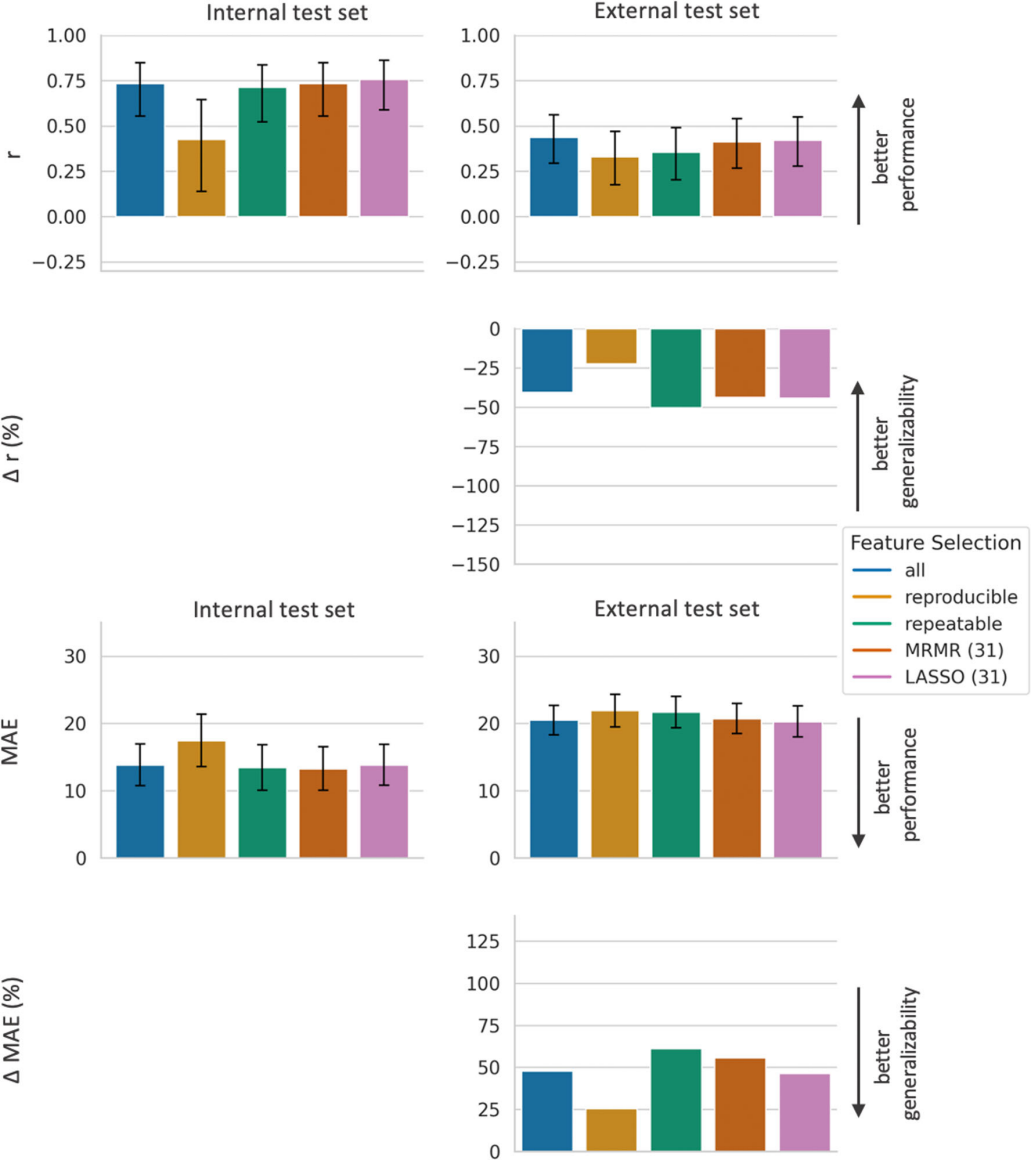
Performance and generalizability of RFR models based on different feature subsets. RFR models based on five different feature subsets, resulting from the five different feature selection approaches (all features, reproducible features, repeatable features, features selected by LASSO, and features selected by MRMR) are compared regarding performance and generalizability. For explanation and abbreviations please see legend of Figure 3. RFR = random forest regressor.

In the first experiment, the influence of the different feature selection approaches on the performance of the radiomics models on the internal test set was investigated, compared with a standard setting in which all radiomics features were used. Results are visualized in the first columns of Figs. 3, 4, 5 for the LR, SVR, and RFR model, respectively. We observed that the performance on the internal data strongly varied across the different ML models: when using all features, *r* ranged from 0.44 for the LR model to 0.73 for the RFR model, and MAE varied from a maximum of 138.0 for the LR model to a minimum of 13.9 for the RFR model.

Using only reproducible features instead of all features resulted for the RFR in a significant decrease of the internal performance, with a decrease in *r* (0.43 vs. 0.73) and an increase in MAE (17.5 vs. 13.9). The LR model benefited from using only reproducible features compared with all features, with an increase in *r* (0.50 vs. 0.44; *P* = 0.77) and a significant decline of MAE (16.6 vs. 138.0). For SVR, there was no marked influence of using only reproducible features compared with using all features on the internal performance (*r* of 0.63 vs. 0.65 (*P* = 0.89) and MAE of 14.4 vs. 14.3 (*P* = 0.59)).

### Influence of Feature Selection on External Performance and Generalizability of Radiomics Models

Performance and generalizability metrics for all combinations of ML models and feature subsets are displayed in Fig. 3 for LR, in Fig. 4 for SVR, and in Fig. 5 for RFR, respectively.

As first general observation, for all models using all features, the performance on the external test set was significantly worse than their performance on the internal test set, with both a significantly lower *r* and a significantly higher MAE. As second general observation, the external performance strongly depended on the ML model: When, for example, models used all features, for the external test set *r* ranged from −0.06 for the LR model to 0.44 for the RFR Model, and MAE ranged from a maximum of 1945.8 for the LR model to a minimum of 20.5 for the RFR Model.

For LR and SVR, the external performance of the model using only reproducible features was superior compared with the model using all features: LR showed a markedly higher *r* (0.14 vs. −0.06; *P* = 0.08) and a significantly lower MAE (25.2 vs. 1946.8), and SVR showed significantly higher *r* (0.43 vs. 0.18) and significantly lower MAE (22.6 vs. 28.2) on the external test set. For RFR, however, on the external test set the model using only reproducible features performed inferior compared with the RFR using all features, with *r* of 0.33 compared with 0.44 (*P* = 0.29) and MAE of 21.9 compared with 20.5 (*P* = 0.10).

When using only reproducible features compared with all features, for all models the generalizability of the model using only reproducible features was better than the generalizability of the model using all features, with a less negative Δ*r* (LR: −72% vs. −115%, SVR: −32% vs. −71%, RFR: −22% vs. −40%), and a smaller ΔMAE (LR: +52% vs. +1310%, SVR: +58% vs. +97%, RFR: +25% vs. +48%).

When using only repeatable features instead of all features, the external performance of the SVR model slightly improved with higher *r* (0.34 vs. 0.18, *P* = 0.16) and lower MAE (27.1 vs. 28.2, *P* = 0.66), while not reaching the external performance of the SVR using only reproducible features with a lower *r* (0.34 vs. 0.43, *P* = 0.38), and a significantly higher MAE (27.1 vs. 22.6). For the RFR, using only repeatable features instead of all features resulted in a lower external performance with lower *r* (0.36 vs. 0.44, *P* = 0.41) and significantly higher MAE (21.7 vs. 20.5), which was similar to the external performance of the RFR using only reproducible features (*r* of 0.36 vs. 0.33, *P* = 0.81, and MAE of 21.7 vs. 21.9, *P* = 0.62).

When comparing the external performance across all combinations of ML models and feature selection methods, a SVR using only reproducible features (*r* = 0.43 and MAE = 22.6), and a RFR using either all features (*r* = 0.44, MAE = 20.5) or a LASSO (*r* = 0.42, MAE = 20.3) or MRMR (*r* = 0.41, MAE = 20.7) as feature selection methods revealed the best external performances.

## Discussion

Despite the vast number of publications in the field of radiomics and despite the apparent success of radiomics models in many single‐center studies continuously published, a decline in performance of radiomics models when applied to external data still prevents radiomics from entering large‐scale clinical application. Several guidelines recommend to isolate robust radiomics features from repeatability and reproducibility studies for feature selection,^14^, ^15^, ^16^, ^17^, ^18^, ^19^ aiming to improve model performance and external generalizability. The current study investigated the influence of a feature selection based on prior in vivo Multi‐MRI‐Scanner reproducibility experiments^9^ on the performance and generalizability of radiomics models on an external, multicentric test set. The results demonstrate that the performance on external, multicentric test data improves for some, but not all machine learning models when using only reproducible features instead of all features. While for LR and SVR the external performance improved, the external performance of the RFR even somewhat declined when using only reproducible features instead of using all features. Across all model‐feature‐selection‐combinations, a RFR using all features showed the best external performance, which was not outperformed by any other combination of ML model and feature selection.

For a radiomics model to be of value for real‐world, multicentric application, both high internal performance and high generalizability are required. Our study design, using both an internal and an external test set, enabled us to separate whether a limited external performance explicitly resulted from a low generalizability, or whether it resulted from a generally low/moderate performance on the internal test set, with or without an additional problem in generalizability. When evaluating the influence of the different feature subsets on generalizability, the results demonstrate that the generalizability for all models benefited from using only reproducible features compared with using all features. For LR and SVR, using only reproducible features instead of all features resulted in a superior external performance. On the other hand, while the RFR using only reproducible features also showed a better generalizability compared with the RFR using all features, this did not translate into a better external performance of the RFR using reproducible features. The RFR using only reproducible features had shown a markedly worse performance on the internal data compared with the RFR using all features, and the benefit in generalizability was outweighed by this effect, resulting in an inferior external performance of the RFR using reproducible features compared with the RFR using all features, despite the superior generalizability. In general, the results demonstrate that the performance of the radiomics models strongly depend on the ML model used for the prediction task, and that additionally the influence which the feature selection has on generalizability of the model also strongly varies across different ML models.

In summary, compared over all model‐feature‐selection‐combinations, a RFR using all features showed the best external performance, which was not outperformed by any other combination of ML model and feature selection. So, against our own expectations and against what might be expected based on several current recommendations on radiomics, an elaborate in vivo multi‐MRI‐scanner test–retest‐study for isolation of reproducible radiomics features did not automatically lead to an improvement of the external performance of the overall best prediction model in the present use case of predicting bone marrow plasma cell infiltration noninvasively from MRI.

While the current study used a subgroup of reproducible features defined in a prior study using ICC as statistical metric and a cutoff of ≥0.8,^9^ it must be stated that there is no unique definition on how to define the subgroup of “stable” radiomics features: different statistical metrics as ICC, concordance correlation coefficient or coefficient of variation and different cutoff‐values have been used in respective prior studies.^4^, ^5^, ^6^, ^7^, ^8^, ^9^, ^10^, ^11^, ^12^, ^13^ Therefore, subsequent studies should evaluate whether different statistical metrics in combination with different cutoff values to select the subgroup of stable radiomics features, or more complex statistical methods integrating feature informativeness and feature stability, might lead to an improvement of the external performance of the RFR model. Further studies should also investigate whether radiomics models based on other MRI sequences or on a multimodal approach can improve the performance on external data.

It seems noteworthy that while the feature subsets selected by LASSO and MRMR include very many texture features, which had not shown to be reproducible across MRI scanners in the earlier study, the external performance of the RFR when using these features is even slightly better compared with the performance when using only reproducible features. This indicates that features that are not well reproducible across scanners nevertheless contribute markedly to the performance of the resulting model, indicating that rather a positive trade‐off of information content to stability than stability of a feature alone is the important characteristic of a radiomics feature to contribute to a radiomics model, which performs (at least relatively) well on external data.

### Limitations

The retrospective study design is a limitation of this study. While the external test set comprised data from eight different MRI scanners (seven different MRI models) from three vendors, it is a limitation of our study that only very few MRIs in the test set have been acquired with 3T, even though the feature reproducibility experiments had included a 3T scanner. While the current study was focused on a regression task, further studies should investigate whether using only reproducible features can improve the external performance of radiomics models for a classification task. As it has been demonstrated that radiomic feature stability can vary between different tumor entities^34^ and must be expected to dependent on imaging modality, further studies across different tumor entities and modalities will be necessary to gain more comprehensive evidence on this topic.

### Conclusion

This study provides evidence on the influence of feature selection based on in‐vivo repeatability and reproducibility experiments on the generalizability and performance of radiomics models on external, multicentric data sets. Using only reproducible radiomics features improved the external performance of some, but not all machine learning models, and did not automatically lead to an improvement of the external performance of the overall best radiomics model. This study highlights the complexity of improving generalizability of radiomics models for multicentric application even when information on Multi‐MRI‐Scanner reproducibility of features is available.

## Supporting information


**Data S1.** Supporting Information.
