# Supplementary material for: Reproducible Radiomics Features from Multi‐MRI‐Scanner Test–Retest‐Study: Influence on Performance and Generalizability of Models
Source: J Magn Reson Imaging. 2024 May 11;61(2):676–86. doi: 10.1002/jmri.29442 (PMC11706307; doi:10.1002/jmri.29442)
Supplement: Supplementary file 1 — Data S1. Supporting Information. [file JMRI-61-676-s001.docx]

**Supplements**

**Additional information on data sets and subject overlap**

Due to the exploratory retrospective nature of this study, there was no formal sample size planning for this study. The training data set was designed to be of reasonable size, especially in comparison to other state-of-the-art radiomics studies, and to be balanced regarding patients with precursor stages and active myeloma, representing the whole biological spectrum of the disease from cases with low to high bone marrow plasma cell infiltration, and all myeloma MRI phenotypes. While the internal test set was similar to the training set regarding scanners, imaging protocols, and tumor load, the external test set deviated in all these properties, as it would often be the case when a radiomics model would be established at one center and then be applied in large-scale clinical practice across different centers with different scanners, imaging protocols, and possible deviations in patient population regarding stage and tumor load, representing a real-world external testing scenario. We report a subject overlap with the following studies: The data set on which the test-retest-experiments were performed had been used to analyze the stability of apparent diffusion coefficient (ADC) - and signal intensity measurements,^1^ stability of size measurements from focal lesions^2^ and repeatability and reproducibility of radiomics features.^3^ The imaging data, biopsy data and segmentations had been used in earlier studies for development of automatic bone marrow segmentation algorithms and automatic prediction of bone marrow biopsy results from MRI,^4–6^ and in large part stems from the prospective, registered ﻿Transregio-79-B8 trial (ClinicalTrials.gov: NCT01374412)^7^ and ﻿GMMG-HD-7 trial (EudraCT: 2017-004768-37).^8^

**Additional information on feature selection**

From the first-order features, volume-and-shape-features, and texture features, which are calculated by the MITK-phenotyping toolbox^9^ (version 2022.4, German Cancer Research Center, Heidelberg, Germany; Git commit hash: 2c898b922f67eb620ddaa0adaf8d518d4430e2d8) per default setting, features were excluded prior to any further feature selection / model training as follows:

1. All volume-and-shape features were excluded, as for this specific task, which is to predict bone marrow biopsy results from segmentations of both hip bones (references for the supplement 4), volume and shape of the hip bones are not expected to carry biological information about the disease.
2. Features which are calculated by MITK Phenotyping under the label “first-order features”, but rather represent volume-and-shape-features or are directly correlated to those, given that we use uniform geometric resampling prior to feature reduction, were excluded: 'First Order Numeric::Number of voxels' and 'First Order Numeric::Sum of voxels'.
3. Features which are calculated by MITK Phenotyping under the label first-order features, but rather represent a quality control than a feature which would potentially carry biological information on the underlying tissue which is analyzed, were also excluded: 'First Order Numeric::Voxel space', 'First Order Numeric::Voxel volume', 'First Order Numeric::Image Dimension', 'First Order Histogram::Number of Bins' and 'First Order Histogram::Bin Size'.

Calculative feature selection was performed on the training data for the radiomics model only to keep all test data sets complete independent from all fitting processes, as incorrect application of feature selection has been demonstrated to lead to highly biased results.^10^

For automatic feature selection, LASSO (scikit-learn 1.3.0) and minimum redundancy maximum relevance (MRMR, pymrmre 1.0.7) were used. The LASSO method was parameterized as follows: maximum number of iterations 50000; optimization tolerance 0.003; regularization parameter 𝛼 0.41 for selecting 31 features. pymrmre was parameterized to produce only a single solution (i.e., no ensembling), for 31 features.

A list of all feature subsets is provided in **Supplementary Tables 1-5**.

**Supplementary Table 1. List of all radiomics features.** Abbreviations: FoF: First-order feature, TF: texture feature, Std.Dev.: standard deviation, Std.: standard, Comb: combined, IVH: intensity volume histogram.

| # | Feature class | # in feature class | Feature name |
| --- | --- | --- | --- |
| 1 | FoF | 1 | 'First Order Numeric::Mean' |
| 2 | FoF | 2 | 'First Order Numeric::Variance' |
| 3 | FoF | 3 | 'First Order Numeric::Skewness' |
| 4 | FoF | 4 | 'First Order Numeric::Excess kurtosis' |
| 5 | FoF | 5 | 'First Order Numeric::Median' |
| 6 | FoF | 6 | 'First Order Numeric::Minimum' |
| 7 | FoF | 7 | 'First Order Numeric::05th Percentile' |
| 8 | FoF | 8 | 'First Order Numeric::10th Percentile' |
| 9 | FoF | 9 | 'First Order Numeric::15th Percentile' |
| 10 | FoF | 10 | 'First Order Numeric::20th Percentile' |
| 11 | FoF | 11 | 'First Order Numeric::25th Percentile' |
| 12 | FoF | 12 | 'First Order Numeric::30th Percentile' |
| 13 | FoF | 13 | 'First Order Numeric::35th Percentile' |
| 14 | FoF | 14 | 'First Order Numeric::40th Percentile' |
| 15 | FoF | 15 | 'First Order Numeric::45th Percentile' |
| 16 | FoF | 16 | 'First Order Numeric::50th Percentile' |
| 17 | FoF | 17 | 'First Order Numeric::55th Percentile' |
| 18 | FoF | 18 | 'First Order Numeric::60th Percentile' |
| 19 | FoF | 19 | 'First Order Numeric::65th Percentile' |
| 20 | FoF | 20 | 'First Order Numeric::70th Percentile' |
| 21 | FoF | 21 | 'First Order Numeric::75th Percentile' |
| 22 | FoF | 22 | 'First Order Numeric::80th Percentile' |
| 23 | FoF | 23 | 'First Order Numeric::85th Percentile' |
| 24 | FoF | 24 | 'First Order Numeric::90th Percentile' |
| 25 | FoF | 25 | 'First Order Numeric::95th Percentile' |
| 26 | FoF | 26 | 'First Order Numeric::Maximum' |
| 27 | FoF | 27 | 'First Order Numeric::Interquantile range' |
| 28 | FoF | 28 | 'First Order Numeric::Range' |
| 29 | FoF | 29 | 'First Order Numeric::Mean absolute deviation' |
| 30 | FoF | 30 | 'First Order Numeric::Robust mean absolute deviation' |
| 31 | FoF | 31 | 'First Order Numeric::Median absolute deviation' |
| 32 | FoF | 32 | 'First Order Numeric::Coefficient of variation' |
| 33 | FoF | 33 | 'First Order Numeric::Quantile coefficient of dispersion' |
| 34 | FoF | 34 | 'First Order Numeric::Energy' |
| 35 | FoF | 35 | 'First Order Numeric::Root mean square' |
| 36 | FoF | 36 | 'First Order Numeric::Standard Deviation' |
| 37 | FoF | 37 | 'First Order Numeric::Kurtosis' |
| 38 | FoF | 38 | 'First Order Numeric::Robust mean' |
| 39 | FoF | 39 | 'First Order Numeric::Robust variance' |
| 40 | FoF | 40 | 'First Order Numeric::Covered image intensity range' |
| 41 | FoF | 41 | 'First Order Numeric::Mode index' |
| 42 | FoF | 42 | 'First Order Numeric::Mode value' |
| 43 | FoF | 43 | 'First Order Numeric::Mode probability' |
| 44 | FoF | 44 | 'First Order Numeric::Entropy' |
| 45 | FoF | 45 | 'First Order Numeric::Uniformtiy' |
| 46 | FoF | 46 | 'First Order Histogram::Mean Value' |
| 47 | FoF | 47 | 'First Order Histogram::Variance Value' |
| 48 | FoF | 48 | 'First Order Histogram::Skewness Value' |
| 49 | FoF | 49 | 'First Order Histogram::Excess Kurtosis Value' |
| 50 | FoF | 50 | 'First Order Histogram::Median Value' |
| 51 | FoF | 51 | 'First Order Histogram::Minimum Value' |
| 52 | FoF | 52 | 'First Order Histogram::Percentile 10 Value' |
| 53 | FoF | 53 | 'First Order Histogram::Percentile 90 Value' |
| 54 | FoF | 54 | 'First Order Histogram::Maximum Value' |
| 55 | FoF | 55 | 'First Order Histogram::Mode Value' |
| 56 | FoF | 56 | 'First Order Histogram::Interquantile Range Value' |
| 57 | FoF | 57 | 'First Order Histogram::Range Value' |
| 58 | FoF | 58 | 'First Order Histogram::Mean Absolute Deviation Value' |
| 59 | FoF | 59 | 'First Order Histogram::Robust Mean Absolute Deviation Value' |
| 60 | FoF | 60 | 'First Order Histogram::Median Absolute Deviation Value' |
| 61 | FoF | 61 | 'First Order Histogram::Coefficient of Variation Value' |
| 62 | FoF | 62 | 'First Order Histogram::Quantile coefficient of Dispersion Value' |
| 63 | FoF | 63 | 'First Order Histogram::Entropy Value' |
| 64 | FoF | 64 | 'First Order Histogram::Uniformity Value' |
| 65 | FoF | 65 | 'First Order Histogram::Robust Mean Value' |
| 66 | FoF | 66 | 'First Order Histogram::Mean Index' |
| 67 | FoF | 67 | 'First Order Histogram::Variance Index' |
| 68 | FoF | 68 | 'First Order Histogram::Skewness Index' |
| 69 | FoF | 69 | 'First Order Histogram::Excess Kurtosis Index' |
| 70 | FoF | 70 | 'First Order Histogram::Median Index' |
| 71 | FoF | 71 | 'First Order Histogram::Minimum Index' |
| 72 | FoF | 72 | 'First Order Histogram::Percentile 10 Index' |
| 73 | FoF | 73 | 'First Order Histogram::Percentile 90 Index' |
| 74 | FoF | 74 | 'First Order Histogram::Maximum Index' |
| 75 | FoF | 75 | 'First Order Histogram::Mode Index' |
| 76 | FoF | 76 | 'First Order Histogram::Interquantile Range Index' |
| 77 | FoF | 77 | 'First Order Histogram::Range Index' |
| 78 | FoF | 78 | 'First Order Histogram::Mean Absolute Deviation Index' |
| 79 | FoF | 79 | 'First Order Histogram::Robust Mean Absolute Deviation Index' |
| 80 | FoF | 80 | 'First Order Histogram::Median Absolute Deviation Index' |
| 81 | FoF | 81 | 'First Order Histogram::Coefficient of Variation Index' |
| 82 | FoF | 82 | 'First Order Histogram::Quantile coefficient of Dispersion Index' |
| 83 | FoF | 83 | 'First Order Histogram::Entropy Index' |
| 84 | FoF | 84 | 'First Order Histogram::Uniformity Index' |
| 85 | FoF | 85 | 'First Order Histogram::Maximum Gradient' |
| 86 | FoF | 86 | 'First Order Histogram::Maximum Gradient Index' |
| 87 | FoF | 87 | 'First Order Histogram::Minimum Gradient' |
| 88 | FoF | 88 | 'First Order Histogram::Minimum Gradient Index' |
| 89 | FoF | 89 | 'First Order Histogram::Robust Mean Index' |
| 90 | TF | 1 | 'Co-occurenced Based Features::Overall Joint Maximum' |
| 91 | TF | 2 | 'Co-occurenced Based Features::Overall Joint Average' |
| 92 | TF | 3 | 'Co-occurenced Based Features::Overall Joint Variance' |
| 93 | TF | 4 | 'Co-occurenced Based Features::Overall Joint Entropy' |
| 94 | TF | 5 | 'Co-occurenced Based Features::Overall Difference Average' |
| 95 | TF | 6 | 'Co-occurenced Based Features::Overall Difference Variance' |
| 96 | TF | 7 | 'Co-occurenced Based Features::Overall Difference Entropy' |
| 97 | TF | 8 | 'Co-occurenced Based Features::Overall Sum Average' |
| 98 | TF | 9 | 'Co-occurenced Based Features::Overall Sum Variance' |
| 99 | TF | 10 | 'Co-occurenced Based Features::Overall Sum Entropy' |
| 100 | TF | 11 | 'Co-occurenced Based Features::Overall Angular Second Moment' |
| 101 | TF | 12 | 'Co-occurenced Based Features::Overall Contrast' |
| 102 | TF | 13 | 'Co-occurenced Based Features::Overall Dissimilarity' |
| 103 | TF | 14 | 'Co-occurenced Based Features::Overall Inverse Difference' |
| 104 | TF | 15 | 'Co-occurenced Based Features::Overall Inverse Difference Normalized' |
| 105 | TF | 16 | 'Co-occurenced Based Features::Overall Inverse Difference Moment' |
| 106 | TF | 17 | 'Co-occurenced Based Features::Overall Inverse Difference Moment Normalized' |
| 107 | TF | 18 | 'Co-occurenced Based Features::Overall Inverse Variance' |
| 108 | TF | 19 | 'Co-occurenced Based Features::Overall Correlation' |
| 109 | TF | 20 | 'Co-occurenced Based Features::Overall Autocorrelation' |
| 110 | TF | 21 | 'Co-occurenced Based Features::Overall Cluster Tendency' |
| 111 | TF | 22 | 'Co-occurenced Based Features::Overall Cluster Shade' |
| 112 | TF | 23 | 'Co-occurenced Based Features::Overall Cluster Prominence' |
| 113 | TF | 24 | 'Co-occurenced Based Features::Overall First Measure of Information Correlation' |
| 114 | TF | 25 | 'Co-occurenced Based Features::Overall Second Measure of Information Correlation' |
| 115 | TF | 26 | 'Co-occurenced Based Features::Overall Row Maximum' |
| 116 | TF | 27 | 'Co-occurenced Based Features::Overall Row Average' |
| 117 | TF | 28 | 'Co-occurenced Based Features::Overall Row Variance' |
| 118 | TF | 29 | 'Co-occurenced Based Features::Overall Row Entropy' |
| 119 | TF | 30 | 'Co-occurenced Based Features::Overall First Row-Column Entropy' |
| 120 | TF | 31 | 'Co-occurenced Based Features::Overall Second Row-Column Entropy' |
| 121 | TF | 32 | 'Co-occurenced Based Features::Mean Joint Maximum' |
| 122 | TF | 33 | 'Co-occurenced Based Features::Mean Joint Average' |
| 123 | TF | 34 | 'Co-occurenced Based Features::Mean Joint Variance' |
| 124 | TF | 35 | 'Co-occurenced Based Features::Mean Joint Entropy' |
| 125 | TF | 36 | 'Co-occurenced Based Features::Mean Difference Average' |
| 126 | TF | 37 | 'Co-occurenced Based Features::Mean Difference Variance' |
| 127 | TF | 38 | 'Co-occurenced Based Features::Mean Difference Entropy' |
| 128 | TF | 39 | 'Co-occurenced Based Features::Mean Sum Average' |
| 129 | TF | 40 | 'Co-occurenced Based Features::Mean Sum Variance' |
| 130 | TF | 41 | 'Co-occurenced Based Features::Mean Sum Entropy' |
| 131 | TF | 42 | 'Co-occurenced Based Features::Mean Angular Second Moment' |
| 132 | TF | 43 | 'Co-occurenced Based Features::Mean Contrast' |
| 133 | TF | 44 | 'Co-occurenced Based Features::Mean Dissimilarity' |
| 134 | TF | 45 | 'Co-occurenced Based Features::Mean Inverse Difference' |
| 135 | TF | 46 | 'Co-occurenced Based Features::Mean Inverse Difference Normalized' |
| 136 | TF | 47 | 'Co-occurenced Based Features::Mean Inverse Difference Moment' |
| 137 | TF | 48 | 'Co-occurenced Based Features::Mean Inverse Difference Moment Normalized' |
| 138 | TF | 49 | 'Co-occurenced Based Features::Mean Inverse Variance' |
| 139 | TF | 50 | 'Co-occurenced Based Features::Mean Correlation' |
| 140 | TF | 51 | 'Co-occurenced Based Features::Mean Autocorrelation' |
| 141 | TF | 52 | 'Co-occurenced Based Features::Mean Cluster Tendency' |
| 142 | TF | 53 | 'Co-occurenced Based Features::Mean Cluster Shade' |
| 143 | TF | 54 | 'Co-occurenced Based Features::Mean Cluster Prominence' |
| 144 | TF | 55 | 'Co-occurenced Based Features::Mean First Measure of Information Correlation' |
| 145 | TF | 56 | 'Co-occurenced Based Features::Mean Second Measure of Information Correlation' |
| 146 | TF | 57 | 'Co-occurenced Based Features::Mean Row Maximum' |
| 147 | TF | 58 | 'Co-occurenced Based Features::Mean Row Average' |
| 148 | TF | 59 | 'Co-occurenced Based Features::Mean Row Variance' |
| 149 | TF | 60 | 'Co-occurenced Based Features::Mean Row Entropy' |
| 150 | TF | 61 | 'Co-occurenced Based Features::Mean First Row-Column Entropy' |
| 151 | TF | 62 | 'Co-occurenced Based Features::Mean Second Row-Column Entropy' |
| 152 | TF | 63 | 'Co-occurenced Based Features::Std.Dev. Joint Maximum' |
| 153 | TF | 64 | 'Co-occurenced Based Features::Std.Dev. Joint Average' |
| 154 | TF | 65 | 'Co-occurenced Based Features::Std.Dev. Joint Variance' |
| 155 | TF | 66 | 'Co-occurenced Based Features::Std.Dev. Joint Entropy' |
| 156 | TF | 67 | 'Co-occurenced Based Features::Std.Dev. Difference Average' |
| 157 | TF | 68 | 'Co-occurenced Based Features::Std.Dev. Difference Variance' |
| 158 | TF | 69 | 'Co-occurenced Based Features::Std.Dev. Difference Entropy' |
| 159 | TF | 70 | 'Co-occurenced Based Features::Std.Dev. Sum Average' |
| 160 | TF | 71 | 'Co-occurenced Based Features::Std.Dev. Sum Variance' |
| 161 | TF | 72 | 'Co-occurenced Based Features::Std.Dev. Sum Entropy' |
| 162 | TF | 73 | 'Co-occurenced Based Features::Std.Dev. Angular Second Moment' |
| 163 | TF | 74 | 'Co-occurenced Based Features::Std.Dev. Contrast' |
| 164 | TF | 75 | 'Co-occurenced Based Features::Std.Dev. Dissimilarity' |
| 165 | TF | 76 | 'Co-occurenced Based Features::Std.Dev. Inverse Difference' |
| 166 | TF | 77 | 'Co-occurenced Based Features::Std.Dev. Inverse Difference Normalized' |
| 167 | TF | 78 | 'Co-occurenced Based Features::Std.Dev. Inverse Difference Moment' |
| 168 | TF | 79 | 'Co-occurenced Based Features::Std.Dev. Inverse Difference Moment Normalized' |
| 169 | TF | 80 | 'Co-occurenced Based Features::Std.Dev. Inverse Variance' |
| 170 | TF | 81 | 'Co-occurenced Based Features::Std.Dev. Correlation' |
| 171 | TF | 82 | 'Co-occurenced Based Features::Std.Dev. Autocorrelation' |
| 172 | TF | 83 | 'Co-occurenced Based Features::Std.Dev. Cluster Tendency' |
| 173 | TF | 84 | 'Co-occurenced Based Features::Std.Dev. Cluster Shade' |
| 174 | TF | 85 | 'Co-occurenced Based Features::Std.Dev. Cluster Prominence' |
| 175 | TF | 86 | 'Co-occurenced Based Features::Std.Dev. First Measure of Information Correlation' |
| 176 | TF | 87 | 'Co-occurenced Based Features::Std.Dev. Second Measure of Information Correlation' |
| 177 | TF | 88 | 'Co-occurenced Based Features::Std.Dev. Row Maximum' |
| 178 | TF | 89 | 'Co-occurenced Based Features::Std.Dev. Row Average' |
| 179 | TF | 90 | 'Co-occurenced Based Features::Std.Dev. Row Variance' |
| 180 | TF | 91 | 'Co-occurenced Based Features::Std.Dev. Row Entropy' |
| 181 | TF | 92 | 'Co-occurenced Based Features::Std.Dev. First Row-Column Entropy' |
| 182 | TF | 93 | 'Co-occurenced Based Features::Std.Dev. Second Row-Column Entropy' |
| 183 | TF | 94 | 'Run Length::Short run emphasis Means' |
| 184 | TF | 95 | 'Run Length::Short run emphasis Std.' |
| 185 | TF | 96 | 'Run Length::Short run emphasis Comb.' |
| 186 | TF | 97 | 'Run Length::Long run emphasis Means' |
| 187 | TF | 98 | 'Run Length::Long run emphasis Std.' |
| 188 | TF | 99 | 'Run Length::Long run emphasis Comb.' |
| 189 | TF | 100 | 'Run Length::Grey level nonuniformity Means' |
| 190 | TF | 101 | 'Run Length::Grey level nonuniformity Std.' |
| 191 | TF | 102 | 'Run Length::Grey level nonuniformity Comb.' |
| 192 | TF | 103 | 'Run Length::Grey level nonuniformity normalized Means' |
| 193 | TF | 104 | 'Run Length::Grey level nonuniformity normalized Std.' |
| 194 | TF | 105 | 'Run Length::Grey level nonuniformity normalized Comb.' |
| 195 | TF | 106 | 'Run Length::Run length nonuniformity Means' |
| 196 | TF | 107 | 'Run Length::Run length nonuniformity Std.' |
| 197 | TF | 108 | 'Run Length::Run length nonuniformity Comb.' |
| 198 | TF | 109 | 'Run Length::Run length nonuniformity normalized Means' |
| 199 | TF | 110 | 'Run Length::Run length nonuniformity normalized Std.' |
| 200 | TF | 111 | 'Run Length::Run length nonuniformity normalized Comb.' |
| 201 | TF | 112 | 'Run Length::Low grey level run emphasis Means' |
| 202 | TF | 113 | 'Run Length::Low grey level run emphasis Std.' |
| 203 | TF | 114 | 'Run Length::Low grey level run emphasis Comb.' |
| 204 | TF | 115 | 'Run Length::High grey level run emphasis Means' |
| 205 | TF | 116 | 'Run Length::High grey level run emphasis Std.' |
| 206 | TF | 117 | 'Run Length::High grey level run emphasis Comb.' |
| 207 | TF | 118 | 'Run Length::Short run low grey level emphasis Means' |
| 208 | TF | 119 | 'Run Length::Short run low grey level emphasis  Std.' |
| 209 | TF | 120 | 'Run Length::Short run low grey level emphasis  Comb.' |
| 210 | TF | 121 | 'Run Length::Short run high grey level emphasis Means' |
| 211 | TF | 122 | 'Run Length::Short run high grey level emphasis Std.' |
| 212 | TF | 123 | 'Run Length::Short run high grey level emphasis Comb.' |
| 213 | TF | 124 | 'Run Length::Long run low grey level emphasis Means' |
| 214 | TF | 125 | 'Run Length::Long run low grey level emphasis Std.' |
| 215 | TF | 126 | 'Run Length::Long run low grey level emphasis Comb.' |
| 216 | TF | 127 | 'Run Length::Long run high grey level emphasis Means' |
| 217 | TF | 128 | 'Run Length::Long run high grey level emphasis Std.' |
| 218 | TF | 129 | 'Run Length::Long run high grey level emphasis Comb.' |
| 219 | TF | 130 | 'Run Length::Run percentage Means' |
| 220 | TF | 131 | 'Run Length::Run percentage Std.' |
| 221 | TF | 132 | 'Run Length::Run percentage Comb.' |
| 222 | TF | 133 | 'Run Length::Number of runs Means' |
| 223 | TF | 134 | 'Run Length::Number of runs Std.' |
| 224 | TF | 135 | 'Run Length::Number of runs Comb.' |
| 225 | TF | 136 | 'Run Length::Grey level variance Means' |
| 226 | TF | 137 | 'Run Length::Grey level variance Std.' |
| 227 | TF | 138 | 'Run Length::Grey level variance Comb.' |
| 228 | TF | 139 | 'Run Length::Run length variance Means' |
| 229 | TF | 140 | 'Run Length::Run length variance Std.' |
| 230 | TF | 141 | 'Run Length::Run length variance Comb.' |
| 231 | TF | 142 | 'Run Length::Run length entropy Means' |
| 232 | TF | 143 | 'Run Length::Run length entropy Std.' |
| 233 | TF | 144 | 'Run Length::Run length entropy Comb.' |
| 234 | TF | 145 | 'Grey Level Size Zone::Small Zone Emphasis' |
| 235 | TF | 146 | 'Grey Level Size Zone::Large Zone Emphasis' |
| 236 | TF | 147 | 'Grey Level Size Zone::Low Grey Level Emphasis' |
| 237 | TF | 148 | 'Grey Level Size Zone::High Grey Level Emphasis' |
| 238 | TF | 149 | 'Grey Level Size Zone::Small Zone Low Grey Level Emphasis' |
| 239 | TF | 150 | 'Grey Level Size Zone::Small Zone High Grey Level Emphasis' |
| 240 | TF | 151 | 'Grey Level Size Zone::Large Zone Low Grey Level Emphasis' |
| 241 | TF | 152 | 'Grey Level Size Zone::Large Zone High Grey Level Emphasis' |
| 242 | TF | 153 | 'Grey Level Size Zone::Grey Level Non-Uniformity' |
| 243 | TF | 154 | 'Grey Level Size Zone::Grey Level Non-Uniformity Normalized' |
| 244 | TF | 155 | 'Grey Level Size Zone::Zone Size Non-Uniformity' |
| 245 | TF | 156 | 'Grey Level Size Zone::Zone Size Non-Uniformity Normalized' |
| 246 | TF | 157 | 'Grey Level Size Zone::Zone Percentage' |
| 247 | TF | 158 | 'Grey Level Size Zone::Grey Level Mean' |
| 248 | TF | 159 | 'Grey Level Size Zone::Grey Level Variance' |
| 249 | TF | 160 | 'Grey Level Size Zone::Zone Size Mean' |
| 250 | TF | 161 | 'Grey Level Size Zone::Zone Size Variance' |
| 251 | TF | 162 | 'Grey Level Size Zone::Zone Size Entropy' |
| 252 | TF | 163 | 'Intensity Volume Histogram::Volume fraction at 0.10 intensity' |
| 253 | TF | 164 | 'Intensity Volume Histogram::Volume fraction at 0.90 intensity' |
| 254 | TF | 165 | 'Intensity Volume Histogram::Intensity at 0.10 volume' |
| 255 | TF | 166 | 'Intensity Volume Histogram::Intensity at 0.90 volume' |
| 256 | TF | 167 | 'Intensity Volume Histogram::Difference volume fraction at 0.10 and 0.90 intensity' |
| 257 | TF | 168 | 'Intensity Volume Histogram::Difference intensity at 0.10 and 0.90 volume' |
| 258 | TF | 169 | 'Intensity Volume Histogram::Area under IVH curve' |

**Supplementary Table 2. List of all reproducible radiomics features.** Abbreviations: FoF: First-order feature, TF: texture feature, Comb: combined.

| # | Feature class | # in feature class | Feature name |
| --- | --- | --- | --- |
| 1 | FoF | 1 | 'First Order Numeric::Mean' |
| 2 | FoF | 2 | 'First Order Numeric::Median' |
| 3 | FoF | 3 | 'First Order Numeric::15th Percentile' |
| 4 | FoF | 4 | 'First Order Numeric::30th Percentile' |
| 5 | FoF | 5 | 'First Order Numeric::35th Percentile' |
| 6 | FoF | 6 | 'First Order Numeric::45th Percentile' |
| 7 | FoF | 7 | 'First Order Numeric::50th Percentile' |
| 8 | FoF | 8 | 'First Order Numeric::55th Percentile' |
| 9 | FoF | 9 | 'First Order Numeric::60th Percentile' |
| 10 | FoF | 10 | 'First Order Numeric::65th Percentile' |
| 11 | FoF | 11 | 'First Order Numeric::70th Percentile' |
| 12 | FoF | 12 | 'First Order Numeric::75th Percentile' |
| 13 | FoF | 13 | 'First Order Numeric::80th Percentile' |
| 14 | FoF | 14 | 'First Order Numeric::85th Percentile' |
| 15 | FoF | 15 | 'First Order Numeric::90th Percentile' |
| 16 | FoF | 16 | 'First Order Numeric::95th Percentile' |
| 17 | FoF | 17 | 'First Order Numeric::Robust mean absolute deviation' |
| 18 | FoF | 18 | 'First Order Numeric::Energy' |
| 19 | FoF | 19 | 'First Order Numeric::Root mean square' |
| 20 | FoF | 20 | 'First Order Numeric::Robust mean' |
| 21 | FoF | 21 | 'First Order Histogram::Mean Value' |
| 22 | FoF | 22 | 'First Order Histogram::Median Value' |
| 23 | FoF | 23 | 'First Order Histogram::Percentile 90 Value' |
| 24 | FoF | 24 | 'First Order Histogram::Robust Mean Absolute Deviation Value' |
| 25 | FoF | 25 | 'First Order Histogram::Robust Mean Value' |
| 26 | TF | 1 | 'Run Length::Run length nonuniformity Means' |
| 27 | TF | 2 | 'Run Length::Run length nonuniformity Comb.' |
| 28 | TF | 3 | 'Run Length::Number of runs Means' |
| 29 | TF | 4 | 'Run Length::Number of runs Comb.' |
| 30 | TF | 5 | 'Intensity Volume Histogram::Volume fraction at 0.90 intensity' |
| 31 | TF | 6 | 'Intensity Volume Histogram::Intensity at 0.10 volume' |

**Supplementary Table 3. List of all repeatable radiomics features.** Abbreviations: FoF: First-order feature, TF: texture feature, Std.Dev.: standard deviation, Std.: standard, Comb: combined, IVH: intensity volume histogram.

| # | Feature class | # in feature class | Feature name |
| --- | --- | --- | --- |
| 1 | FoF | 1 | First Order Numeric::Mean |
| 2 | FoF | 2 | First Order Numeric::Variance |
| 3 | FoF | 3 | First Order Numeric::Skewness |
| 4 | FoF | 4 | First Order Numeric::Median |
| 5 | FoF | 5 | First Order Numeric::05th Percentile |
| 6 | FoF | 6 | First Order Numeric::10th Percentile |
| 7 | FoF | 7 | First Order Numeric::15th Percentile |
| 8 | FoF | 8 | First Order Numeric::20th Percentile |
| 9 | FoF | 9 | First Order Numeric::25th Percentile |
| 10 | FoF | 10 | First Order Numeric::30th Percentile |
| 11 | FoF | 11 | First Order Numeric::35th Percentile |
| 12 | FoF | 12 | First Order Numeric::40th Percentile |
| 13 | FoF | 13 | First Order Numeric::45th Percentile |
| 14 | FoF | 14 | First Order Numeric::50th Percentile |
| 15 | FoF | 15 | First Order Numeric::55th Percentile |
| 16 | FoF | 16 | First Order Numeric::60th Percentile |
| 17 | FoF | 17 | First Order Numeric::65th Percentile |
| 18 | FoF | 18 | First Order Numeric::70th Percentile |
| 19 | FoF | 19 | First Order Numeric::75th Percentile |
| 20 | FoF | 20 | First Order Numeric::80th Percentile |
| 21 | FoF | 21 | First Order Numeric::85th Percentile |
| 22 | FoF | 22 | First Order Numeric::90th Percentile |
| 23 | FoF | 23 | First Order Numeric::95th Percentile |
| 24 | FoF | 24 | First Order Numeric::Maximum |
| 25 | FoF | 25 | First Order Numeric::Interquantile range |
| 26 | FoF | 26 | First Order Numeric::Mean absolute deviation |
| 27 | FoF | 27 | First Order Numeric::Robust mean absolute deviation |
| 28 | FoF | 28 | First Order Numeric::Median absolute deviation |
| 29 | FoF | 29 | First Order Numeric::Coefficient of variation |
| 30 | FoF | 30 | First Order Numeric::Quantile coefficient of dispersion |
| 31 | FoF | 31 | First Order Numeric::Energy |
| 32 | FoF | 32 | First Order Numeric::Root mean square |
| 33 | FoF | 33 | First Order Numeric::Standard Deviation |
| 34 | FoF | 34 | First Order Numeric::Robust mean |
| 35 | FoF | 35 | First Order Numeric::Robust variance |
| 36 | FoF | 36 | First Order Numeric::Mode value |
| 37 | FoF | 37 | First Order Histogram::Mean Value |
| 38 | FoF | 38 | First Order Histogram::Variance Value |
| 39 | FoF | 39 | First Order Histogram::Skewness Value |
| 40 | FoF | 40 | First Order Histogram::Median Value |
| 41 | FoF | 41 | First Order Histogram::Percentile 10 Value |
| 42 | FoF | 42 | First Order Histogram::Percentile 90 Value |
| 43 | FoF | 43 | First Order Histogram::Maximum Value |
| 44 | FoF | 44 | First Order Histogram::Mode Value |
| 45 | FoF | 45 | First Order Histogram::Interquantile Range Value |
| 46 | FoF | 46 | First Order Histogram::Mean Absolute Deviation Value |
| 47 | FoF | 47 | First Order Histogram::Robust Mean Absolute Deviation Value |
| 48 | FoF | 48 | First Order Histogram::Median Absolute Deviation Value |
| 49 | FoF | 49 | First Order Histogram::Coefficient of Variation Value |
| 50 | FoF | 50 | First Order Histogram::Quantile coefficient of Dispersion Value |
| 51 | FoF | 51 | First Order Histogram::Robust Mean Value |
| 52 | FoF | 52 | First Order Histogram::Skewness Index |
| 53 | FoF | 53 | First Order Histogram::Coefficient of Variation Index |
| 54 | FoF | 54 | First Order Histogram::Quantile coefficient of Dispersion Index |
| 55 | TF | 1 | Co-occurenced Based Features::Overall Correlation |
| 56 | TF | 2 | Co-occurenced Based Features::Overall First Measure of Information Correlation |
| 57 | TF | 3 | Co-occurenced Based Features::Overall Second Measure of Information Correlation |
| 58 | TF | 4 | Co-occurenced Based Features::Mean Correlation |
| 59 | TF | 5 | Co-occurenced Based Features::Mean First Measure of Information Correlation |
| 60 | TF | 6 | Co-occurenced Based Features::Mean Second Measure of Information Correlation |
| 61 | TF | 7 | Co-occurenced Based Features::Std.Dev. Joint Entropy |
| 62 | TF | 8 | Co-occurenced Based Features::Std.Dev. Difference Entropy |
| 63 | TF | 9 | Co-occurenced Based Features::Std.Dev. Sum Entropy |
| 64 | TF | 10 | Co-occurenced Based Features::Std.Dev. Correlation |
| 65 | TF | 11 | Co-occurenced Based Features::Std.Dev. Second Measure of Information Correlation |
| 66 | TF | 12 | Run Length::Grey level nonuniformity normalized Std. |
| 67 | TF | 13 | Run Length::Run length nonuniformity Means |
| 68 | TF | 14 | Run Length::Run length nonuniformity Comb. |
| 69 | TF | 15 | Run Length::Number of runs Means |
| 70 | TF | 16 | Run Length::Number of runs Comb. |
| 71 | TF | 17 | Intensity Volume Histogram::Volume fraction at 0.90 intensity |
| 72 | TF | 18 | Intensity Volume Histogram::Intensity at 0.10 volume |
| 73 | TF | 19 | Intensity Volume Histogram::Intensity at 0.90 volume |
| 74 | TF | 20 | Intensity Volume Histogram::Difference intensity at 0.10 and 0.90 volume |
| 75 | TF | 21 | Grey Level Size Zone::Grey Level Non-Uniformity |

**Supplementary Table 4. List of 31 radiomics features selected by LASSO algorithm.** Abbreviations: FoF: First-order feature, TF: texture feature, Std.Dev.: standard deviation, Std.: standard.

| # | Feature class | # in feature class | Feature name |
| --- | --- | --- | --- |
| 1 | FoF | 1 | 'First Order Numeric::Variance' |
| 2 | FoF | 2 | 'First Order Numeric::Excess kurtosis' |
| 3 | FoF | 3 | 'First Order Numeric::95th Percentile' |
| 4 | FoF | 4 | 'First Order Numeric::Interquantile range' |
| 5 | FoF | 5 | 'First Order Numeric::Range' |
| 6 | FoF | 6 | 'First Order Numeric::Standard Deviation' |
| 7 | FoF | 7 | 'First Order Numeric::Covered image intensity range' |
| 8 | FoF | 8 | 'First Order Histogram::Excess Kurtosis Value' |
| 9 | FoF | 9 | 'First Order Histogram::Range Value' |
| 10 | FoF | 10 | 'First Order Histogram::Range Index' |
| 11 | FoF | 11 | 'First Order Histogram::Maximum Gradient' |
| 12 | FoF | 12 | 'First Order Histogram::Minimum Gradient Index' |
| 13 | TF | 1 | 'Intensity Volume Histogram::Volume fraction at 0.10 intensity' |
| 14 | TF | 2 | 'Intensity Volume Histogram::Difference volume fraction at 0.10 and 0.90 intensity' |
| 15 | TF | 3 | 'Co-occurenced Based Features::Overall Cluster Shade' |
| 16 | TF | 4 | 'Co-occurenced Based Features::Mean Cluster Shade' |
| 17 | TF | 5 | 'Co-occurenced Based Features::Std.Dev. Joint Maximum' |
| 18 | TF | 6 | 'Co-occurenced Based Features::Std.Dev. Joint Variance' |
| 19 | TF | 7 | 'Co-occurenced Based Features::Std.Dev. Joint Entropy' |
| 20 | TF | 8 | 'Co-occurenced Based Features::Std.Dev. Sum Entropy' |
| 21 | TF | 9 | 'Co-occurenced Based Features::Std.Dev. Cluster Shade' |
| 22 | TF | 10 | 'Co-occurenced Based Features::Std.Dev. Row Maximum' |
| 23 | TF | 11 | 'Co-occurenced Based Features::Std.Dev. Row Variance' |
| 24 | TF | 12 | 'Run Length::Long run low grey level emphasis Means' |
| 25 | TF | 13 | 'Run Length::Long run low grey level emphasis Std.' |
| 26 | TF | 14 | 'Run Length::Long run high grey level emphasis Std.' |
| 27 | TF | 15 | 'Run Length::Grey level variance Std.' |
| 28 | TF | 16 | 'Run Length::Run length variance Means' |
| 29 | TF | 17 | 'Grey Level Size Zone::Large Zone Low Grey Level Emphasis' |
| 30 | TF | 18 | 'Grey Level Size Zone::Grey Level Non-Uniformity' |
| 31 | TF | 19 | 'Grey Level Size Zone::Zone Size Entropy' |

**Supplementary Table 5. List of 31 radiomics features selected by MRMR algorithm.** Abbreviations: FoF: First-order feature, TF: texture feature, Std.Dev.: standard deviation, Std.: standard, Comb: combined.

| # | Feature class | # in feature class | Feature name |
| --- | --- | --- | --- |
| 1 | FoF | 1 | 'First Order Numeric::20th Percentile' |
| 2 | FoF | 2 | 'First Order Numeric::Covered image intensity range' |
| 3 | FoF | 3 | 'First Order Numeric::Kurtosis' |
| 4 | FoF | 4 | 'First Order Numeric::Quantile coefficient of dispersion' |
| 5 | FoF | 5 | 'First Order Numeric::Skewness' |
| 6 | FoF | 6 | 'First Order Numeric::Minimum' |
| 7 | FoF | 7 | 'First Order Numeric::Robust variance' |
| 8 | FoF | 8 | 'First Order Histogram::Maximum Value' |
| 9 | FoF | 9 | 'First Order Histogram::Maximum Gradient' |
| 10 | TF | 1 | 'Co-occurenced Based Features::Std.Dev. Inverse Difference Moment' |
| 11 | TF | 2 | 'Co-occurenced Based Features::Std.Dev. Difference Entropy' |
| 12 | TF | 3 | 'Co-occurenced Based Features::Overall Cluster Shade' |
| 13 | TF | 4 | 'Co-occurenced Based Features::Std.Dev. First Measure of Information Correlation' |
| 14 | TF | 5 | 'Co-occurenced Based Features::Std.Dev. Joint Entropy' |
| 15 | TF | 6 | 'Co-occurenced Based Features::Std.Dev. Row Entropy' |
| 16 | TF | 7 | 'Co-occurenced Based Features::Std.Dev. Sum Entropy' |
| 17 | TF | 8 | 'Co-occurenced Based Features::Std.Dev. Inverse Difference' |
| 18 | TF | 9 | 'Co-occurenced Based Features::Std.Dev. Sum Average' |
| 19 | TF | 10 | 'Co-occurenced Based Features::Std.Dev. Row Maximum' |
| 20 | TF | 11 | 'Co-occurenced Based Features::Std.Dev. Cluster Prominence' |
| 21 | TF | 12 | 'Co-occurenced Based Features::Std.Dev. Inverse Variance' |
| 22 | TF | 13 | 'Co-occurenced Based Features::Mean First Measure of Information Correlation' |
| 23 | TF | 14 | 'Run Length::Number of runs Means' |
| 24 | TF | 15 | 'Run Length::Short run high grey level emphasis Std.' |
| 25 | TF | 16 | 'Run Length::Low grey level run emphasis Std.' |
| 26 | TF | 17 | 'Run Length::Long run high grey level emphasis Std.' |
| 27 | TF | 18 | 'Run Length::Grey level nonuniformity Comb.' |
| 28 | TF | 19 | 'Run Length::High grey level run emphasis Std.' |
| 29 | TF | 20 | 'Run Length::Grey level variance Std.' |
| 30 | TF | 21 | 'Grey Level Size Zone::Grey Level Non-Uniformity' |
| 31 | TF | 22 | 'Grey Level Size Zone::Large Zone Low Grey Level Emphasis' |

## Further details on machine learning

We chose to include several different ML models for prediction of the target variable, to account for the fact that the ML model might influence both the absolute performance and the generalizability of the model, therefore being a potential confounder for investigating the influence of the feature selection on performance and generalizability. The models were selected because they are prototypical for their classes and their different levels of inherent regularization. As a baseline, linear regressor was used due to its simplicity and minimal set of trainable parameters.

As a more complex nonlinear approach, we used support vector regressor with a radial basis function kernel. Since it is a typical model employed in conjunction with radiomics features, random forest regressor was used as third model, which is based on ensemble learning and has an inherent regularization of constraining its parameter number ^11^.

Radiomics features from the training set were normalized by removing the mean from the respective feature (calculated based on the training set) and subsequently scaling to unit variance, using scikit-learn StandardScaler. Radiomics features from samples in the test set underwent the identical transformation.

No hyperparameter optimization was performed for LR. SVR and RFR model hyperparameters were optimized using 5-fold cross-validation on the training data and a grid search optimizing the mean squared error. Supplementary Table 6 reports the parameter grids used for optimizing the individual methods.

**Supplementary Table 6. Parameter grids used for optimization of support vector regressor and random forest regressor.**

| Model | Parameter grid |
| --- | --- |
| Support vector regressor | 'C': [0.1, 1, 10, 100, 1000]  'gamma': [1, 0.1, 0.01, 0.001, 0.0001] |
| Random forest regressor | 'max_depth': [2, 4, 6]  'n_estimators': [500] |

The following software packages were used: scipy 1.11.1, numpy 1.25.0, pandas 2.0.3, seaborn 0.12.2, matplotlib 3.7.1, sklearn 1.3.0, joblib 1.3.1, pymrmre 1.0.7, python 3.10.12.

**References**

1. Wennmann M, Thierjung H, Bauer F, et al. Repeatability and Reproducibility of ADC Measurements and MRI Signal Intensity Measurements of Bone Marrow in Monoclonal Plasma Cell Disorders. *Invest. Radiol.* 2022;57(4):272–281. Available at: https://journals.lww.com/investigativeradiology/Fulltext/2022/04000/Repeatability_and_Reproducibility_of_ADC.8.aspx.

2. Wennmann M, Grözinger M, Weru V, et al. Test–retest, inter- and intra-rater reproducibility of size measurements of focal bone marrow lesions in MRI in patients with multiple myeloma. *Br. J. Radiol.* 2023;96(1145). Available at: https://www.birpublications.org/doi/10.1259/bjr.20220745.

3. Wennmann M, Bauer F, Klein A, et al. In Vivo Repeatability and Multiscanner Reproducibility of MRI Radiomics Features in Patients With Monoclonal Plasma Cell Disorders. *Invest. Radiol.* 2023;58(4):253–264. Available at: https://journals.lww.com/investigativeradiology/Fulltext/2023/04000/In_Vivo_Repeatability_and_Multiscanner.2.aspx.

4. Wennmann M, Ming W, Bauer F, et al. Prediction of Bone Marrow Biopsy Results From MRI in Multiple Myeloma Patients Using Deep Learning and Radiomics. *Invest. Radiol.* 2023;58(10):754–765. Available at: https://journals.lww.com/10.1097/RLI.0000000000000986.

5. Wennmann M, Klein A, Bauer F, et al. Combining Deep Learning and Radiomics for Automated, Objective, Comprehensive Bone Marrow Characterization From Whole-Body MRI. *Invest. Radiol.* 2022;57(11):752–763. Available at: https://journals.lww.com/investigativeradiology/Fulltext/9900/Combining_Deep_Learning_and_Radiomics_for.23.aspx.

6. Wennmann M, Neher P, Stanczyk N, et al. Deep Learning for Automatic Bone Marrow Apparent Diffusion Coefficient Measurements From Whole-Body Magnetic Resonance Imaging in Patients With Multiple Myeloma. *Invest. Radiol.* 2023;58(4):273–282. Available at: https://journals.lww.com/10.1097/RLI.0000000000000932.

7. Wennmann M, Goldschmidt H, Mosebach J, et al. Whole-body magnetic resonance imaging plus serological follow-up for early identification of progression in smouldering myeloma patients to prevent development of end-organ damage. *Br. J. Haematol.* 2022;199(1):65–75. Available at: https://doi.org/10.1111/bjh.18232.

8. Goldschmidt H, Mai EK, Bertsch U, et al. Addition of isatuximab to lenalidomide, bortezomib, and dexamethasone as induction therapy for newly diagnosed, transplantation-eligible patients with multiple myeloma (GMMG-HD7): part 1 of an open-label, multicentre, randomised, active-controlled, phase. *Lancet. Haematol.* 2022;9(11):e810–e821.

9. Götz M, Nolden M, Maier-Hein K. MITK Phenotyping: An open-source toolchain for image-based personalized medicine with radiomics. *Radiother. Oncol.* 2019;131:108–111. Available at: http://www.sciencedirect.com/science/article/pii/S0167814018336156.

10. Demircioğlu A. Measuring the bias of incorrect application of feature selection when using cross-validation in radiomics. *Insights Imaging*. 2021;12(1):172.

11. Mentch L, Zhou S. Randomization as Regularization: A Degrees of Freedom Explanation for Random Forest Success. *J. Mach. Learn. Res.* 2019;21:1–36. Available at: http://arxiv.org/abs/1911.00190.
